# The Post-mating Switch in the Pheromone Response of *Nasonia* Females Is Mediated by Dopamine and Can Be Reversed by Appetitive Learning

**DOI:** 10.3389/fnbeh.2018.00014

**Published:** 2018-01-30

**Authors:** Maria Lenschow, Michael Cordel, Tamara Pokorny, Magdalena M. Mair, John Hofferberth, Joachim Ruther

**Affiliations:** ^1^Institute of Zoology, University of Regensburg, Regensburg, Germany; ^2^Department of Chemistry, Kenyon College, Gambier, OH, United States

**Keywords:** appetitive learning, dopamine, neuromodulator, octopamine, olfactory plasticity

## Abstract

The olfactory sense is of crucial importance for animals, but their response to chemical stimuli is plastic and depends on their physiological state and prior experience. In many insect species, mating status influences the response to sex pheromones, but the underlying neuromodulatory mechanisms are poorly understood. After mating, females of the parasitic wasp *Nasonia vitripennis* are no longer attracted to the male sex pheromone. Here we show that this post-mating behavioral switch is mediated by dopamine (DA). Females fed a DA-receptor antagonist prior to mating maintained their attraction to the male pheromone after mating while virgin females injected with DA became unresponsive. However, the switch is reversible as mated females regained their pheromone preference after appetitive learning. Feeding mated *N. vitripennis* females with antagonists of either octopamine- (OA) or DA-receptors prevented relearning of the pheromone preference suggesting that both receptors are involved in appetitive learning. Moreover, DA injection into mated females was sufficient to mimic the oviposition reward during odor conditioning with the male pheromone. Our data indicate that DA plays a key role in the plastic pheromone response of *N. vitripennis* females and reveal some striking parallels between insects and mammals in the neuromodulatory mechanisms underlying olfactory plasticity.

## Introduction

Survival and reproductive success of animals are often closely associated with their ability to use chemical information. Accurate chemical sensing by animals is crucial for fundamental processes such as locating food and mates or avoiding natural enemies and suboptimal environmental conditions (Petrulis, 2013; Suh et al., 2014; Wyatt, 2014). However, the maintenance of a functional and highly sensitive olfactory system to detect informative odors that trigger beneficial behavioral responses implies metabolic and ecological costs and thus, it is adaptive to respond to chemical stimuli only in certain contexts. As a consequence, olfaction is typically plastic and the behavioral responses of animals to chemical stimuli are often restricted to situations in which they are beneficial (Palmer and Kristan, 2011; Bonzano et al., 2016; Gadenne et al., 2016).

Olfactory plasticity has been extensively studied in insects, for which the use of chemical information is indispensable. The male sex pheromone response in many moth species, for instance, follows a circadian rhythm ensuring a maximal response in phases of female pheromone calling (Castrovillo and Carde, 1979; Haynes and Birch, 1984; Linn et al., 1996). Other important factors influencing the strength of odor guided behavior in insects are related to their physiological state (Gadenne et al., 2016). Starving individuals, for example, respond more strongly to food-related odors than satiated ones (Edgecomb et al., 1994; Reisenman et al., 2013; Reisenman, 2014) and the response to sex pheromones depends on the age insects reach sexual maturity (Shorey et al., 1968; Landolt and Heath, 1988). Similar to some mammals (Serguera et al., 2008), the mating status of insects is another intrinsic parameter modulating their olfactory response to sex pheromones (Gadenne et al., 2016). Females of many insect species mate only once in their lifetime and males, despite being mostly polygamous, are often abstinent between copulations to replenish their sperm reservoir. Consequently, insects often show a transient or permanent post-mating switch in their olfactory preferences, i.e., the preference for sex pheromones is paused or replaced by a preference for food and oviposition related odors (Jang, 1995; Gadenne et al., 2001; Steiner and Ruther, 2009a; Saveer et al., 2012; Kromann et al., 2015). Newly mated males of the moth *Agrotis ipsilon*, for instance, are completely unresponsive to the female sex pheromone. This behavioral switch is correlated with a decreased sensitivity of interneurons in the antennal lobe whereas the antennal sensitivity is unaffected (Gadenne et al., 2001). In *Spodoptera littoralis*, another moth species with a post-mating behavioral switch in the male sex pheromone response, the switch is characterized by decreased sensitivity of both pheromone detecting sensilla and the respective glomeruli in the antennal lobe (Kromann et al., 2015).

In addition to plasticity modulated by circadian rhythms and physiological state, experience based plasticity is common in insect olfaction (Dukas, 2008). Despite their relatively simple brain structure, insects have the ability to learn odors associatively with rewards such as sugar, mating or oviposition success (appetitive learning) as well as with punishments such as electric shocks or unpalatable food (aversive learning). They may store this information in short- and long-term memory from which it can be retrieved to optimize future responses to the conditioned odors (Giurfa, 2013, 2015; Perry and Barron, 2013).

The biochemical and neuromodulatory mechanisms underlying the plasticity of insect olfaction have been extensively studied in the past decades. It has become clear that neuromodulators from different chemical classes interact at different levels of the olfactory system and are responsible for the variability of behavioral responses of insects to chemical stimuli (Gadenne et al., 2016). Biogenic amines such as dopamine (DA), octopamine (OA) and serotonin, hormones such as juvenile hormone, 20-hydroxyecdysone or insulin and neuropeptides such as Aea-HP-I from *Aedes aegypti* or sNPF from *Drosophila melanogaster* have been studied in this respect (reviewed by Gadenne et al., 2016). The target receptors of plasticity-related neuromodulators can be located in both the peripheral (olfactory sensory neurons, OSN) and the central nervous system (antennal lobe, mushroom bodies and lateral horn; Gadenne et al., 2016). Mechanistic studies addressing the post-mating behavioral switch of pheromone responses have hitherto focused on male moths as well as on female fruitflies (Jang, 1995, 2002) and parasitic wasps (Ruther et al., 2007, 2010; Ruther and Hammerl, 2014). The post-mating behavioral switch in females of the Mediterranean fruitfly *Ceratitis capitata* is mediated by undefined substances from the male accessory gland that are transferred to the female during copulation (Jang, 1995, 2002). After mating or injection of accessory gland extracts, females no longer respond to the male sex pheromone. In *Drosophila melanogaster* males, unsuccessful courtship with unreceptive females leads to enhanced pheromone-based discrimination against mated females which is mediated by DA (Keleman et al., 2012).

While in mammals DA releasing neurons have been shown to modulate post-mating olfactory preferences (Serguera et al., 2008), the neuromodulators underlying the post-mating switch in the pheromone response of insects are still unknown. Candidates such as OA and serotonin (Barrozo et al., 2010) as well as 20-hydroxyecdysone (Vitecek et al., 2013) have been tested with male *A. ipsilon* moths but were found to be behaviorally inactive. As for aversive learning, numerous studies on different taxa including fruit flies (Schwaerzel et al., 2003), honey bees (Vergoz et al., 2007) and crickets (Unoki et al., 2005; Matsumoto et al., 2015; Awata et al., 2016), have shown that DA is of central importance. In *D. melanogaster*, opto- and thermogenetic approaches have been used to locate DA releasing neurons that are involved in aversive learning. These neurons are located in the two neuron clusters PPTL1 and PAM of the mushroom bodies, the storage site of olfactory memories (Claridge-Chang et al., 2009; Aso et al., 2010; Waddell, 2013). Studies on *D. melanogaster* (Kim et al., 2007) and the cricket *Gryllus bimaculatus* (Awata et al., 2015, 2016) have revealed that the type 1 DA receptors dDA1 (*D. melanogaster*) and Dop1 (*G. bimaculatus*) are involved in aversive learning in these species. As for appetitive learning, there appear to be species-related differences in the role of biogenic amines as neuromodulators. Studies addressing the role of biogenic amines in appetitive learning of honeybees and crickets suggested that OA release is crucial and sufficient for appetitive learning (Hammer, 1993; Hammer and Menzel, 1998; Unoki et al., 2005; Awata et al., 2015, 2016; Matsumoto et al., 2015) thus pointing to contrasting roles of OA and DA in appetitive and aversive learning, respectively. This scenario was long assumed to also be valid for *D. melanogaster* (Schwaerzel et al., 2003), but recent work has demonstrated that the role of DA is not restricted to aversive learning and both DA and OA act in a concerted manner during appetitive learning via the receptors dDA1 and OAMB, respectively (Kim et al., 2007, 2013; Burke et al., 2012; Liu et al., 2012; Waddell, 2013). Hence, additional species need to be studied to get a clearer picture of the role of OA and DA in appetitive learning.

In the present study, we investigate the neuromodulatory mechanisms of the post-mating behavioral switch and appetitive learning in *Nasonia vitripennis*, a pupal parasitoid of numerous fly species and a model organism for the study of parasitic wasp biology (Whiting, 1967; Werren et al., 2010). Virgin females of this species are attracted by a substrate-borne sex attractant which is produced in the rectal vesicle of the males and deposited through the anal orifice (Ruther et al., 2007; Abdel-Latief et al., 2008; Steiner and Ruther, 2009b). The pheromone consists of the three components (4*R*,5*S*)-5-hydroxy-4-decanolide (*RS*), (4*R*,5*R*)-5-hydroxy-4-decanolide (*RR*) and 4-methylquinazoline (4-MQ; Ruther et al., 2007, 2008). After mating, females are no longer attracted by the male pheromone and prefer host odors instead. In some studies, mated females even avoided the male pheromone (Ruther et al., 2007; Steiner and Ruther, 2009a). This behavioral switch appears to be permanent since mated females remain unresponsive at least 6 days after mating (Ruther et al., 2007). Detailed behavioral analyses revealed that the switch is independent of sperm transfer. Rather, it correlates with the receptivity signal shown by the female in response to the male pre-mating courtship behavior (Ruther et al., 2010). A decisive element of this courtship is the so-called head nodding behavior by the male after mounting the female (Van Den Assem et al., 1980; Ruther et al., 2010). Male head nodding serves to release a secretion from an oral gland which has two effects on the female. First, the secretion elicits the receptivity signal in the female, but the pheromone components responsible for this effect are still unknown (Ruther, 2013). Second, the secretion terminates the female response to the male abdominal sex pheromone (Van Den Assem et al., 1980; Ruther et al., 2010). The chemicals mediating the second effect have been recently identified as three fatty acid ethyl esters (Ruther and Hammerl, 2014).

Using a pharmacological approach, we aim here to take the next step toward a full understanding of the post-mating behavioral switch in *N. vitripennis* females by investigating the underlying neuromodulatory mechanism. In particular, we investigated whether the switch is mediated by DA and/or OA. We furthermore studied whether the post-mating behavioral switch is “hard-wired” or can be principally reversed by appetitive learning and, if so, whether OA, DA or both neuromodulators are involved in this process.

## Materials and Methods

### Insects

The *N. vitripennis* used in this study originated from the inbred strain Phero01, which was reared on freeze-killed puparia of the green bottle fly *Lucilia caesar* as described previously (Steiner et al., 2006). To obtain virgin wasps of defined age, parasitoid pupae were excised from host puparia 1–2 days prior to eclosion and kept singly in 1.5 ml microcentrifuge tubes until emergence. Mated females were obtained by putting a virgin female and a male in an observation chamber until copulation occurred.

### General Procedures for the Behavioral Bioassays

The response of differently treated females to the synthetic sex pheromone was examined using a static four-chamber olfactometer made of acrylic glass (Steidle and Schöller, 1997; Ruther and Steidle, 2000). The olfactometer consisted of a cylinder (4 cm high, 19 cm diameter) that was divided by crosswise-arranged vertical plates into four chambers of equal size. Two opposing chambers were equipped with round glass dishes (5.5 cm diameter, 2 cm height) that were turned upside down for sample presentation, the other two chambers were left empty and defined as neutral zones. A walking arena (19 cm diameter) consisting of fine metal gauze and a rim of acrylic glass (10 mm height) was placed on the cylinder. The olfactometer was illuminated from above with a desk lamp (60 W). For each test, 2 μl of a dichloromethane solution of the synthetic pheromone (200 ng/μl *RS*, 100 ng/μl *RR* and 3 ng/μl 4-MQ), synthesized as described previously (Ruther et al., 2008, 2016) were applied to a disk of filter paper. The ratio of the pheromone components is consistent with the previously reported composition (Ruther et al., 2007, 2008). Control paper disks were treated with pure dichloromethane. After evaporation of the solvent, test and control disks were put onto the bottom of the reversed glass dishes and, after mounting the walking arena, females were released individually into its center. The time females spent in the arena sectors above the test and control chambers was recorded for 5 min using The Observer XT observational software (Noldus, Wageningen, Netherlands). Female parasitoids were used only once and the pheromone was renewed after every replicate (*n* = 20 per treatment). The arena was cleaned after every 3–4 replicates with ethanol and distilled water and dried before being used again. All experiments were performed between noon and 6 p.m. To avoid biased results due to side preferences of the parasitoids, the olfactometer was turned clockwise by 90° after each test.

### Appetitive Learning in Mated Females

**Figure 1:**
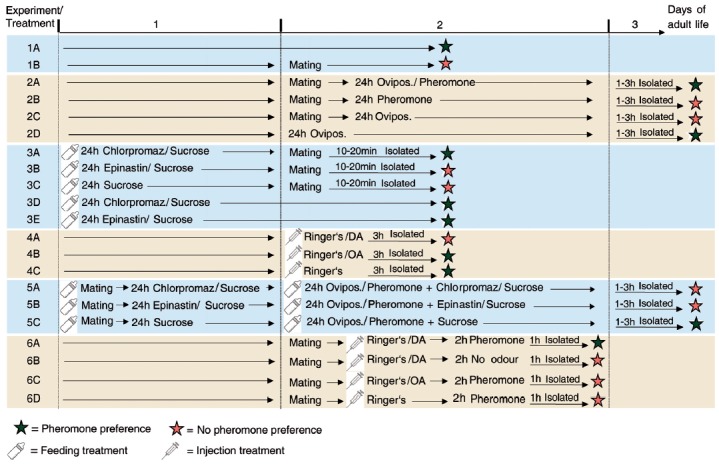
Overview of the experiments performed. Schematic view and chronological order of the treatments of *N. vitripennis* females of the different experiments performed in this study.

This series of experiments was performed to investigate whether the behavioral switch of mated females is “hard-wired” and thus irreversible or whether mated females may principally regain responsiveness to the male pheromone after appetitive learning. Like most parasitic wasps, *N. vitripennis* females have the capability to learn odors associatively during oviposition (Schurmann et al., 2009, 2012, 2015; Hoedjes et al., 2015) which was therefore used as a reward in our experiment. In classical Pavlovian conditioning experiments, wasps are exposed to a conditioned stimulus (here: odor) in the presence of an unconditioned stimulus (here: host) and subsequently show a conditioned response (here: preference for the odor) in the absence of the unconditioned stimulus. Before the conditioning experiment, we verified in Experiment 1 that the experimental set-up was suitable to establish pheromone preference and the post-mating behavioral switch in females by investigating the response of virgin and mated females (Experiment 1, treatments A-B, a schematic protocol for all experiments is given in Figure 1). In Experiment 2, mated females were conditioned for 24 h by offering them three hosts for oviposition (in 1.5 ml microcentrifuge tubes) in the presence of 1 μg of synthetic male pheromone (applied to a filter paper disk, ratio as described above, treatment 2A). In a previous learning study on *Nasonia*, this conditioning paradigm was found to be sufficient for the formation of long term memory (Schurmann et al., 2012). For control, we exposed mated females for 24 h to either the pheromone in absence of the hosts (treatment 2B) or to hosts in absence of the pheromone (treatment 2C). Finally, we exposed virgin females to hosts for 24 h. Virgin females readily oviposit like mated ones producing, however, only male offspring. Thus, this treatment allowed conclusions whether oviposition *per se* influences the pheromone response (treatment 2D). In all these treatments, females were subsequently isolated for 1–3 h in microcentrifuge tubes and tested in the olfactometer bioassay as described above.

### Role of Neuromodulators in the Post-mating Behavioral Switch

To investigate whether DA and/or OA are involved in the post-mating behavioral switch of females, we fed females in Experiment 3 prior to mating with 10% (m/v) sucrose solutions containing either the DA receptor antagonist chlorpromazine, the OA receptor antagonist epinastine (100 μg/ml each, Sigma-Aldrich) or no additional compound (control). Chlorpromazine and epinastine have been used previously in similar pharmacological studies on insect learning (Unoki et al., 2005; Matsumoto et al., 2015). Doses of 75 μl of the solutions were applied into 0.2-ml microcentrifuge tubes and a small plug of cotton wool was pushed into the tube until it was soaked with the solutions. Subsequently, females were put into the tubes and allowed to lick the solutions from the soaked cotton wool for 24 h. Subsequently, females were mated (receptor antagonist feeding did neither influence mating behavior nor frequency), isolated for 10–20 min and tested in the olfactometer bioassay (treatments 3A-C). To eliminate the possibility that the antagonists influence the pheromone response of females *per se*, we also fed virgin females with chlorpromazine or epinastine solutions as described above and tested them without prior mating (treatments 3D-E). Since our feeding experiments did not allow for control of the active antagonist dose taken up by the wasps, we chose a second approach in experiment 4 in which we injected the agonists (DA and OA) into virgin *N. vitripennis* females to directly test their role in the behavioral switch. We injected virgin females into the abdominal tip with 69 nl of freshly prepared Ringer’s solutions (Yan et al., 2016) containing 19 ng of either DA or OA (Sigma-Aldrich) using a Nanoliter 2010 microinjector mounted to a micromanipulator (World Precision Instruments; treatments 4A-B). Doses of injected neuromodulators were adapted from previously published data for honey bees (Scheiner et al., 2002) after body mass correction. Control females were injected with pure Ringer’s solution (treatment 4C). After injection, females were isolated for 3 h in microcentrifuge tubes and tested in the pheromone bioassay as described above.

### Role of Neuromodulators in Appetitive Learning of Mated Females

To investigate whether DA and/or OA are involved in the appetitive learning of the male pheromone by mated females, we fed in Experiment 5 mated females for 24 h with sucrose solutions of chlorpromazine, epinastine or pure sucrose solution (control) as described above. Subsequently, females were conditioned for 24 h by exposing them to hosts and the male pheromone as described above. During conditioning, females still had access to the receptor antagonist solutions and the sucrose solution provided during the initial 24 h feeding. After conditioning, females were tested in the pheromone bioassay (treatment 5A-C). To directly test the role of DA and OA in appetitive learning of *N. vitripennis* females, we injected mated females in experiment 6 with DA, OA and pure Ringer’s solution (control) as described above and exposed them (in 1.5 ml glass vials) to 1 μg of the synthetic pheromone for 2 h. Subsequently, females were kept isolated for another hour in empty microcentrifuge tubes and tested in the pheromone assay as described above (treatments 6A, C, D). In this experiment, injected females had access to neither hosts nor sucrose solution during pheromone exposure and thus it allowed conclusions as to whether appetitive learning can be simulated by replacing the reward through DA or OA injection. Given that DA injection had this effect (see “Results” section) we performed another control experiment to test whether DA injection alone re-establishes the pheromone preference of mated females. For this purpose, we injected mated females with DA and kept them isolated for 2 h in the absence of the pheromone (treatment 6B).

### Statistical Analyses

Residence times of females in the arena sectors above the test and control chambers were compared for each treatment by a Wilcoxon matched-pairs tests using Past 3.0 scientific software (Hammer et al., 2001). Furthermore, we performed a *post hoc* statistical power analysis based on the effect sizes (Cohens’s dz) from treatments with significant pheromone preference, and considering an α-error of 0.05 and *n* = 20 replicates using G-power 3.1.9.2 scientific software (Faul et al., 2007; see Supplementary Tables S1–S6 in the electronic supplementary material). Effect sizes of significant results were large (>0.93), resulting in a statistical power of >0.97, which exceeds the recommended minimum value of 0.8 (Cohen, 1992). Hence, this analysis allowed for conclusions on whether differently treated females preferred, avoided or responded indifferently to the male sex pheromone. Differences between treatments within experiments were analyzed in R (R Development Core Team, 2015) by fitting a generalized linear model (GLM) assuming pseudo-binomial error structure (i.e., with estimated dispersion parameter) and logit link function. The proportion of time spent in the pheromone field was used as response variable, treatment as fixed factor and the total time spent in both fields (pheromone plus control) as weights (Faraway, 2016). Selective pairwise comparisons were subsequently performed using the same procedure, followed by Benjamini-Hochberg correction to control for multiple testing (see Supplementary Table S7 in the electronic supplementary material).

## Results

### Mated Females Regain the Preference for the Male Sex Pheromone by Appetitive Learning

**Figure 2:**
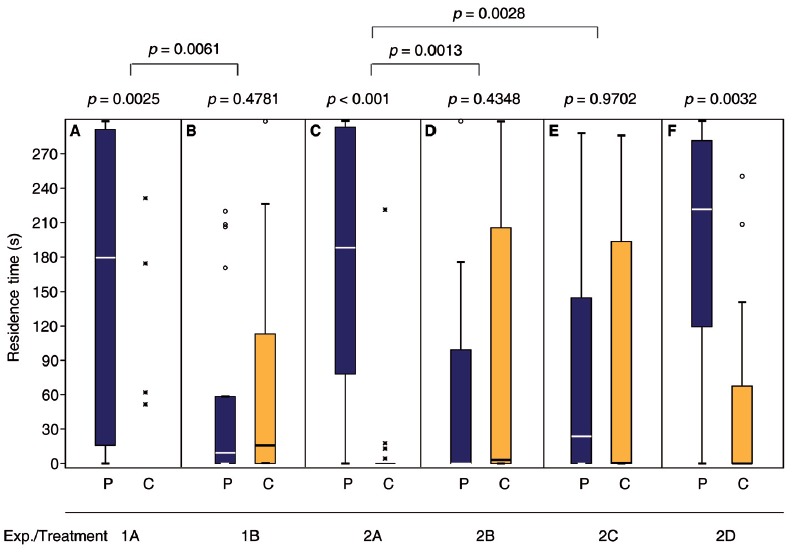
Mated females regain the preference for the male sex pheromone by appetitive learning. Given are the residence times of differently treated *N. vitripennis* females in the two odor fields of a static two-choice olfactometer when given the choice between the synthetic male sex pheromone (P) and a solvent control (C): **(A)** Virgin females; **(B)** mated females; mated females exposed for 24 h to **(C)** oviposition reward + pheromone, **(D)** oviposition reward alone, or **(E)** pheromone alone; **(F)** virgin females exposed for 24 h to oviposition reward. Exact treatments for each experiment are given in Figure 1. Box-and-whisker plots show median (horizontal line), 25%–75% quartiles (box), maximum/minimum range (whiskers) and outliers (° >1.5× above box height; * >3× above box height; data analysis within treatments by Wilcoxon matched-pairs tests and between treatments by generalized linear models (GLM) *n* = 20).

Consistent with our previous findings (Ruther et al., 2007, 2010; Steiner and Ruther, 2009a), the response of *N. vitripennis* females to the male sex pheromone depended on their mating status. Only virgin females were attracted and, after mating, they no longer responded to the male pheromone (Figures 2A,B, Supplementary Tables S1 and S7 in the electronic supplementary material). Mated females that were given the opportunity to lay eggs into hosts in the presence of the male pheromone became responsive again and preferred the pheromone in subsequent bioassays (Figure 2C, Supplementary Tables S2, S7). However, when mated females were exposed to either the male pheromone or hosts alone (Figures 2D,E) they did not regain the preference for the male pheromone. A 24 h oviposition period did not influence the attraction of virgin females to the male sex pheromone (Figure 2F).

### DA but Not OA Is Involved in the Post-mating Behavioral Switch of *N. Vitripennis* Females

**Figure 3:**
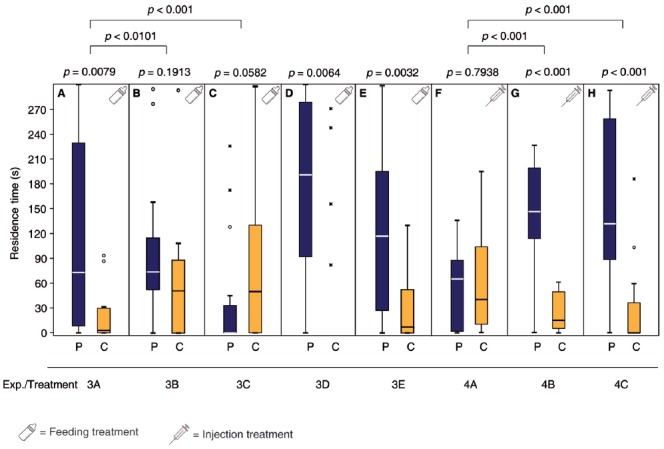
Dopamine (DA) but not octopamine (OA) is involved in the post-mating behavioral switch of *N. vitripennis* females. Given are the residence times of differently treated *N. vitripennis* females in the two odor fields of a static two-choice olfactometer when given the choice between the synthetic male sex pheromone (P) and a solvent control (C): Mated females fed prior to mating for 24 h **(A)** chlorpromazine/sucrose, **(B)** epinastine/sucrose, or **(C)** only sucrose; virgin females fed for 24 h **(D)** chlorpromazine/sucrose or **(E)** epinastine/sucrose; virgin females injected with **(F)** DA, **(G)** OA, or **(H)** Ringer’s solution. Exact treatments for each experiment are given in Figure 1. Box-and-whisker plots show median (horizontal line), 25%–75% quartiles (box), maximum/minimum range (whiskers) and outliers (° >1.5× above box height; * >3× above box height; data analysis within treatments by Wilcoxon matched-pairs tests and between treatments by GLM; *n* = 20).

Mated females fed a sucrose solution containing the DA antagonist chlorpromazine over 24 h prior to mating did not show the behavioral switch and still responded to the male pheromone (Figure 3A, Supplementary Tables S3, S7) while the OA antagonist epinastine did not have this effect (Figure 3B). Mated control females, fed with pure sucrose solution prior to mating showed a non-significant tendency to avoid the male pheromone (Figure 3C). Neither chlorpromazine nor epinastine feeding influenced the pheromone response of virgin females (Figures 3D,E). Injection of DA into the abdomen made virgin females unresponsive to the male sex pheromone whereas OA and pure Ringer’s solution did not have this effect (Figures 3F–H, Supplementary Tables S4, S7).

### Both DA and OA Are Involved in Appetitive Learning of Mated *N. Vitripennis* Females

**Figure 4:**
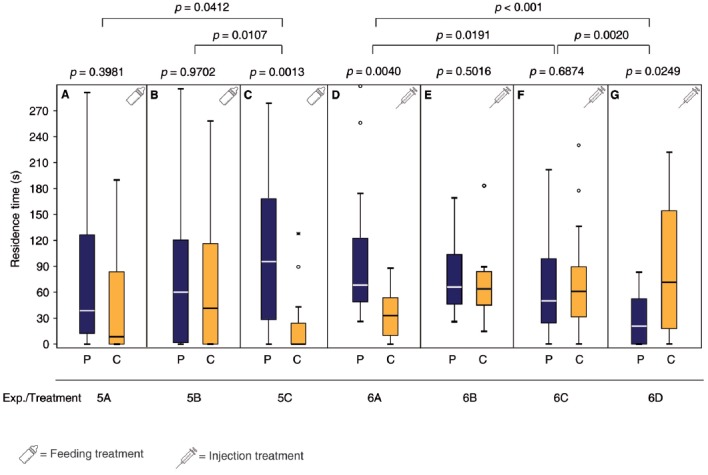
Both DA and OA are involved in appetitive learning of mated *N. vitripennis* females. Given are the residence times of differently treated *N. vitripennis* females in the two odor fields of a static two-choice olfactometer when given the choice between the synthetic male sex pheromone (P) and a solvent control (C): Mated females exposed for 24 h to oviposition reward + pheromone after having been fed for 24 h **(A)** chlorpromazine/sucrose, **(B)** epinastine/sucrose or **(C)** only sucrose; mated females exposed for 2 h to the pheromone after having been injected with **(D)** DA, **(F)** OA, or **(G)** Ringer’s solution; **(E)** mated females exposed for 2 h to no stimulus after having been injected with DA. Exact treatments for each experiment are given in Figure 1. Box-and-whisker plots show median (horizontal line), 25%–75% quartiles (box), maximum/minimum range (whiskers) and outliers (° >1.5× above box height; * >3× above box height; data analysis within treatments by Wilcoxon matched-pairs tests and between treatments by GLM; *n* = 20).

Females fed after mating for 24 h with a sucrose solution containing the DA antagonist chlorpromazine or the OA antagonist epinastine did not regain the pheromone preference after oviposition in the presence of the pheromone (Figures 4A,B, Supplementary Table S5). In contrast, mated control females fed with a pure sucrose solution did regain the pheromone preference (Figure 4C). Mated females injected with Ringer’s solution and then exposed to the male pheromone, avoided the pheromone in the subsequent pheromone assay (Figure 4G, Supplementary Tables S6, S7) while this avoidance was absent in mated females injected with OA prior to pheromone exposure (Figure 4F). In contrast, mated females injected with DA prior to pheromone exposure preferred the male pheromone in the subsequent pheromone assay (Figure 4D). However, DA injection without pheromone exposure was not sufficient to re-establish the pheromone preference of mated females (Figure 4E).

## Discussion

The present study is an important step in unraveling the neuromodulatory mechanism underlying the post-mating switch in the pheromone response of *N. vitripennis*. Feeding virgin females with the DA antagonist chlorpromazine prior to mating prevented the switch while injection of DA into virgin females made them unresponsive to the male sex pheromone. This suggests that DA release is responsible for the lost pheromone preference in mated *N. vitripennis* females. It remains to be investigated whether this switch is specific for the pheromone or whether also the response to host odors is modified. The fact that mated females prefer host volatiles over the pheromone (Steiner and Ruther, 2009a) suggests that DA release might have opposing effects on the olfactory response to the pheromone and host odor, respectively.

Given the well-established role of DA in aversive learning, it is tempting to assume that the behavioral switch is a result of aversive learning, i.e., females are conditioned during courtship by perceiving the male pheromone and subsequently avoid the male odor. However, we can exclude this scenario because: (a) in a previous study, females that had never been exposed to the male pheromone were “switched-off” after mating (Ruther et al., 2010); and (b) in this study, DA injection in the absence of the pheromone was sufficient to elicit the behavioral switch.

A DA-mediated modulatory effect on an insect’s pheromone response caused by previous sexual contact has been recently demonstrated in *D. melanogaster* (Keleman et al., 2012). After unsuccessful courtship with unreceptive females, males display enhanced discrimination against mated females in subsequent encounters, thereby using the male-derived sex pheromone cis-vaccenyl acetate (cVA) as a cue. This compound is transferred to the female during copulation and thus indicates mating refractoriness of the respective females. Similar to the post-mating switch in *Nasonia*, this so-called “courtship learning” does not require the presence of the stimulus during the unsuccessful courtship and is mediated by dopaminergic neurons. In *Drosophila*, these neurons provide input to the mushroom bodies via the DA receptor DopR1 (Keleman et al., 2012).

An important question arising from our data is whether DA has similar effects in other insects showing post-mating behavioral plasticity. A recent study on *A. ipsilon* suggests that this is not the case, because males injected with DA were significantly more responsive to the female sex pheromone than control males injected with Ringer’s solution (Abrieux et al., 2014). Provided that male and female insects respond similarly to DA, this result is in contrast to the present study. Hence, more insect species need to be investigated before more general conclusions can be drawn. The increased responsivenes in *A. ipsilon* is mediated via the G-protein-coupled receptor DopEcR which also binds to the insect hormone ecdysone (Abrieux et al., 2013, 2014). The DA receptor involved in the post-mating behavioral switch of *N. vitripennis* females is unknown. The fact that we found an inibitory effect of chlorpromazine, an antagonist of D1- and D2-like DA receptors (Giannini et al., 1984), suggests that one of these receptor types is involved.

Unlike the post-mating olfactory switch in female fruit flies (Jang, 1995, 2002), the behavioral switch in *N. vitripennis* females is independent of the transfer of a male ejaculate (Ruther et al., 2010). Rather, it is elicited by an oral pheromone released by the males to the female antennae during courtship (Ruther and Hammerl, 2014). This suggests that the post-mating switch is the result of a male strategy to monopolize females by decreasing the chance of recently mated females to encounter other males. The chemicals eliciting the post-mating olfactory switch in *N. vitripennis* females have been identified as the three fatty acid esters, ethyl oleate, ethyl linoleate and ethyl linolenate. Antennal contact with these esters makes females unresponsive to the male abdominal sex attractant (Ruther and Hammerl, 2014). A challenging task of future research will be the identification and localization of the neuronal circuits linking the perception of these esters with the DA release in the females’ brain.

Our results demonstrate that the post-mating behavioral switch of *N. vitripennis* females is not “hard-wired”. Rather, the pheromone preference can be re-acquired by appetitive learning using oviposition as a reward. It is unlikely that this occurs in nature, because *N. vitripennis* females typically disperse shortly after mating to search for new oviposition sites while the brachypterous males are unable to follow (Whiting, 1967; Ruther et al., 2014). Thus, females are unlikely to oviposit in the presence of the male sex pheromone in nature. Nevertheless, our experiments show that the peripheral elements of the olfactory system underlying sex pheromone perception are still functional after mating and can be used for appetitive relearning of the pheromone. Previous studies have shown that *N. vitripennis* ovipositing females can be conditioned with a wide array of odors including spices or anthropogenic substances including those that they are unlikely to encounter in a natural environment (Schurmann et al., 2009, 2012). Consequently, it is unlikely that the regained preference of mated females for the male pheromone comes along with an increased interest in remating. Rather, it seems plausible that they are using the relearned pheromone as a cue in the context of foraging for oviposition sites.

Our study does not support contrasting roles of OA and DA in appetitive and aversion learning, respectively. Rather, the results of our receptor antagonist experiments support a concerted function of OA and DA in appetitive learning in *N. vitripennis* as has been shown in *D. melanogaster*. In this species, a subset of dopaminergic neurons has been identified in the mushroom bodies that are located downstream of octopaminergic neurons and are necessary for appetitive learning (Kim et al., 2007; Burke et al., 2012; Liu et al., 2012). In our experiments, feeding of both OA and DA receptor antagonists prior to odor conditioning prevented appetitive learning. Moreover, direct injection of DA into mated females was sufficient to mimic the oviposition reward during pheromone conditioning (Figure 4D). OA injection, however, did not suffice to mimic the reward, but OA injected females did not avoid the pheromone whereas Ringer-injected control females did (Figures 4F,G).

Previous studies suggest that DA is not involved in appetitive learning in all insects but *D. melanogaster*. In crickets, different DA receptor antagonists impaired only aversive learning while OA receptor antagonists impaired appetitive learning (Unoki et al., 2005; Matsumoto et al., 2015), and knock-out of the DA receptor dop1 impaired aversive but not appetitive learning (Awata et al., 2015). In honeybees, DA injection has even been shown to impair appetitive memory consolidation (Klappenbach et al., 2013).

The demonstrated crucial role of DA in both the behavioral switch and appetitive learning in *N. vitripennis* implies intriguing parallels between insects and mammals. A DA-modulated post-mating switch of olfactory responses to male odor has been shown previously in mice (Serguera et al., 2008). Copulation elicits a surge of DA in the main olfactory bulb of female mice which impairs the perception of volatile pheromones contained in male urine. Compared to *Nasonia* females, however, this post-mating switch occurs after a long delay (3 days after copulation). Pregnant female mice exposed to the smell of a foreign male’s urine will suffer abortion (Bruce effect). After the behavioral switch, however, females are no longer sensitive to the smell and thus will avoid terminating their pregnancy (Serguera et al., 2008).

Apart from mediating post-mating behavioral plasticity, DA is also involved in appetitive learning in mammals. Numerous studies have demonstrated that reward learning depends on intact DA function and rewards are typically rendered ineffective in animals that have had their DA systems blocked by DA antagonists (Harley, 2004; Wise, 2004; Yue et al., 2004; Schultz, 2013). This suggests a convergent evolution of the neuromodulatory mechanisms underlying olfactory plasticity in insects and mammals which deserves further research.

## Author Contributions

JR conceived the study. ML, MC and TP collected the data. ML, MC, TP and MMM analyzed the data. JH provided the synthetic (*4R*,5*S*)-5-hydroxy-4-decanolide. JR wrote the manuscript. ML, MC, TP, MMM and JH reviewed the manuscript.

## Conflict of Interest Statement

The authors declare that the research was conducted in the absence of any commercial or financial relationships that could be construed as a potential conflict of interest. The reviewer DB and handling Editor declared their shared affiliation, and the handling Editor states that the process nevertheless met the standards of a fair and objective review.
