# Supplementary material for: The Post-mating Switch in the Pheromone Response of Nasonia Females Is Mediated by Dopamine and Can Be Reversed by Appetitive Learning
Source: Front Behav Neurosci. 2018 Jan 30;12:14. doi: 10.3389/fnbeh.2018.00014 (PMC5797616; doi:10.3389/fnbeh.2018.00014)
Supplement: Supplementary file 1 [file Table_1.pdf]

Supplementary Material

The post-mating switch in the pheromone response of *Nasonia* females is mediated by dopamine and can be reversed by appetitive learning

Maria Lenschow[1]. Michael Cordel[1]. Tamara Pokorny[1], Magdalena M. Mair[1]. John Hofferberth[2]. Joachim Ruther[1*]

[1]Institute of Zoology. University of Regensburg. Regensburg. Germany

[2]Department of Chemistry. Kenyon College. Gambier. Ohio. USA

***Correspondence:**
joachim.ruther@ur.de

1 Supplementary Tables

Supplementary Table S1. Residence times (s) in experiment 1 of differently treated *N. vitripennis* females in the two odour fields of a static two-choice olfactometer when given the choice between the synthetic male sex pheromone and a solvent control. The exact description of the different treatments is given in Fig.1 of the main paper.

	Treatment 1A		Treatment 1B	
Wasp no.	Pheromone	Control	Pheromone	Control
1	298.5	0	170.45	2.15
2	0	51.7	0	226.24
3	144.3	0	0	70.66
4	137.21	0	2.66	0
5	4.98	174.32	21.68	132.91
6	3.48	0	0	298.31
7	291.08	0	16.48	0
8	130.53	0	9.4	86.5
9	298.49	0	16.94	0
10	179.46	61.98	0	112.93
11	298.5	0	36.27	0
12	216.52	0	208.43	0
13	247.31	0	58.5	133.4
14	298.3	0	0	1.38
15	264.75	0	14.57	15.81
16	187.43	0	0	77.28
17	15.84	231.29	4.84	26.21
18	0	0	206.12	0
19	210.03	0	1.58	21.18
20	178.36	0	219.71	0
Mean	170.3	26.0	49.4	60.2
Standard deviation	112.3	63.6	79.7	84.5
Cohen's dz	1.4794		0.13138	
Statistical power	**0.99998**		0.08457	

Supplementary Table S2. Residence times (s) in experiment 2 of differently treated *N. vitripennis* females in the two odour fields of a static two-choice olfactometer when given the choice between the synthetic male sex pheromone and a solvent control. The exact description of the different treatments is given in Fig.1 of the main paper.

	Treatment 2A		Treatment 2B		Treatment 2C		Treatment 2D	
Wasp no.	Pheromone	Control	Pheromone	Control	Pheromone	Control	Pheromone	Control
1	188.08	0	0	17.6	259.43	0.55	191.86	30.47
2	7.49	17.7	0	268.44	106.11	0	119.52	140.83
3	26.43	12.98	0	0	0	252.07	298.7	0
4	293.33	0	98.01	0	46.46	0	221.56	0
5	298.7	0	0	0	0	10.77	298.7	0
6	244.82	0	0	4.29	0	164.56	119.47	0.63
7	293.31	0	0	205.45	144.5	0	121.75	60.77
8	264.33	0	0	0	288.09	0	79.43	67.57
9	157.79	0	0	114.98	215.68	0	298.64	0
10	263.33	0	0	275.47	23.79	58.37	0	208.5
11	298.7	0	99.09	0	0	286.17	0	250.35
12	273.3	0	0	298.42	82.48	0	279.43	0
13	164.6	0	151.1	0	198.42	0	19.23	140.41
14	77.97	0	0	3.18	0	172.67	266.85	0
15	36.79	4.29	175.73	0	9.47	201.24	298.7	0
16	293.67	0	298.46	0	109.7	0	162.44	0
17	298.7	0	108.33	0	0	2.98	221.73	0
18	100.61	0	0	297.78	0	220.39	265.05	0
19	0	221.34	0	75.71	15.06	193.65	281.5	0
20	78.18	0	8.42	5.9	111.93	0	266.55	0
Mean	183.0	12.8	47.0	78.4	80.6	78.2	190.6	45.0
Standard deviation	112.8	49.3	82.7	118.0	95.4	105.2	105.6	77.6
Cohen's dz	1.73770		0.29932		0.00238		1.53659	
Statistical power	**1.00000**		0.23624		0.05001		**0.99999**	

Supplementary Table S3. Residence times (s) in experiment 3 of differently treated *N. vitripennis* females in the two odour fields of a static two-choice olfactometer when given the choice between the synthetic male sex pheromone and a solvent control. The exact description of the different treatments is given in Fig.1 of the main paper.

	Treatment 3A		Treatment 3B		Treatment 3C		Treatment 3D		Treatment 3E	
Wasp no.	Pheromone	Control	Pheromone	Control	Pheromone	Control	Pheromone	Control	Pheromone	Control
1	293.1	0.0	37.8	93.0	0.0	32.1	241.5	0.0	150.5	0.0
2	5.6	93.5	58.1	58.0	0.0	195.2	247.0	0.0	228.3	0.0
3	14.0	86.7	72.6	92.6	0.0	0.0	0.0	271.2	27.5	0.0
4	0.0	0.0	115.5	84.9	2.3	26.9	228.3	0.0	141.1	0.0
5	73.2	0.0	55.7	75.1	3.7	55.0	2.3	0.0	118.0	11.1
6	0.0	28.4	114.1	33.0	0.0	292.4	112.1	81.9	31.7	70.3
7	215.0	31.5	78.0	108.8	45.0	81.7	164.8	0.0	155.4	0.0
8	209.7	0.0	74.2	51.2	7.0	130.3	22.9	155.8	0.0	91.7
9	8.5	0.0	148.9	0.0	0.0	0.0	299.6	0.0	195.7	71.5
10	91.5	4.7	46.8	88.5	0.0	89.7	263.0	0.0	0.0	4.8
11	229.8	0.0	296.0	0.0	33.1	11.7	191.1	0.0	131.4	33.2
12	129.6	2.9	158.8	25.7	0.0	297.8	279.2	0.0	20.6	130.5
13	3.6	9.0	52.8	81.5	128.1	59.4	299.7	0.0	270.6	0.0
14	14.0	31.0	95.2	0.0	27.1	0.0	239.7	0.0	82.0	35.0
15	299.7	0.0	77.9	28.3	226.0	0.0	298.1	0.0	13.5	38.4
16	56.8	29.9	278.1	0.0	0.0	30.7	0.0	248.2	299.4	0.0
17	12.6	5.6	89.1	40.6	0.0	50.0	299.7	0.0	181.1	52.7
18	111.2	27.3	0.0	294.5	0.0	55.3	185.7	0.0	233.2	6.9
19	286.9	0.0	12.8	0.0	172.5	0.0	92.1	0.0	60.4	0.0
20	299.6	0.0	65.2	87.2	0.0	290.3	178.5	0.0	108.8	26.9
Mean	117.7	17.5	96.4	62.1	32.2	84.9	182.3	37.9	122.5	28.7
Standard deviation	117.1	27.8	76.1	66.4	65.0	102.3	108.0	85.1	92.6	37.4
Cohen's dz	0.94554		0.47809		0.58771		1.46501		1.16253	
Statistical power	**0.97408**		0.50724		0.68099		**0.99998**		**0.99771**	

Supplementary Table S4. Residence times (s) in experiment 4 of differently treated *N. vitripennis* females in the two odour fields of a static two-choice olfactometer when given the choice between the synthetic male sex pheromone and a solvent control. The exact description of the different treatments is given in Fig.1 of the main paper.

	Treatment 4*a*		Treatment 4*b*		Treatment 4c	
Wasp no.	Pheromone	Control	Pheromone	Control	Pheromone	Control
1	0.0	173.4	220.8	6.8	286.8	0.0
2	75.2	0.0	75.5	5.2	253.3	0.0
3	0.0	97.6	98.1	20.8	54.3	103.4
4	55.4	42.4	146.6	49.8	132.0	0.0
5	0.0	194.9	0.0	0.0	270.9	0.0
6	1.6	161.7	199.4	3.8	156.8	0.0
7	135.8	42.7	114.1	7.5	96.6	59.8
8	0.0	183.4	218.5	6.7	117.5	0.0
9	65.1	88.7	193.0	0.0	19.5	0.0
10	123.3	18.8	98.4	15.2	137.0	0.0
11	79.6	10.2	131.2	5.8	103.6	0.0
12	65.1	7.1	150.1	61.5	267.0	0.0
13	64.9	104.0	122.8	50.8	239.9	36.7
14	16.7	0.0	139.3	31.0	153.5	0.0
15	75.9	26.2	186.7	26.6	98.9	36.3
16	92.0	40.1	227.0	2.5	88.8	11.8
17	127.1	37.1	219.5	17.9	41.8	185.9
18	73.1	54.6	189.1	27.8	259.0	0.0
19	44.6	0.0	116.1	56.9	0.0	0.0
20	87.5	32.7	182.5	56.0	293.5	0.0
Mean	59.1	65.8	151.4	22.6	153.5	21.7
Standard deviation	44.2	65.4	58.8	21.3	95.0	47.1
Cohen's dz	0.16784		2.4999		1.60198	
Statistical power	0.10695		**1.00000**		**1.00000**	

Supplementary Table S5. Residence times (s) in experiment 5 of differently treated *N. vitripennis* females in the two odour fields of a static two-choice olfactometer when given the choice between the synthetic male sex pheromone and a solvent control. The exact description of the different treatments is given in Fig.1 of the main paper.

	Treatment 5*a*		Treatment 5*b*		Treatment 5*c*	
Wasp no.	Pheromone	Control	Pheromone	Control	Pheromone	Control
1	12.6	8.6	0.0	67.5	268.2	22.3
2	69.1	28.2	38.5	39.2	0.0	89.4
3	4.3	72.1	97.9	67.4	0.0	0.0
4	112.0	0.0	36.7	111.8	92.4	1.5
5	126.5	1.8	109.0	58.2	91.2	0.0
6	21.0	57.7	80.1	105.2	243.5	0.0
7	38.5	190.0	103.9	80.1	0.0	24.2
8	262.8	0.0	79.4	84.6	87.5	0.0
9	45.2	122.7	140.9	38.6	95.6	0.0
10	147.1	0.0	78.7	22.3	138.5	0.0
11	0.0	129.0	8.1	83.1	85.5	43.2
12	291.2	0.0	39.6	20.1	122.7	0.0
13	58.1	35.7	210.5	27.7	244.8	0.0
14	0.0	0.0	7.3	7.0	108.6	127.8
15	0.0	4.8	88.3	45.9	28.3	24.7
16	75.6	83.7	193.3	0.0	138.1	0.0
17	38.7	36.3	20.8	55.5	278.1	0.0
18	20.8	3.8	115.2	40.9	123.9	13.2
19	219.3	1.9	169.0	25.1	168.1	0.0
20	36.5	100.4	60.8	69.5	1.4	1.6
Mean	79.0	43.8	83.9	52.5	115.8	17.4
Standard deviation	88.5	55.5	60.8	31.2	89.1	34.0
Cohen's dz	0.45440		0.59627		1.26347	
Statistical power	0.46827		0.69351		**0.99942**	

Supplementary Table S6. Residence times (s) in experiment 6 of differently treated *N. vitripennis* females in the two odour fields of a static two-choice olfactometer when given the choice between the synthetic male sex pheromone and a solvent control. The exact description of the different treatments is given in Fig.1 of the main paper.

	Treatment 6A		Treatment 6B		Treatment 6C		Treatment 6D	
Wasp no.	Pheromone	Control	Pheromone	Control	Pheromone	Control	Pheromone	Control
1	123.1	9.1	61.9	69.5	201.8	6.2	2.4	195.5
2	53.3	49.5	28.2	90.7	105.5	105.6	2.3	115.6
3	47.5	67.7	45.9	85.6	44.5	50.2	0.0	0.0
4	72.8	53.8	67.3	70.6	78.7	57.6	34.5	17.6
5	48.7	41.8	33.8	184.4	50.2	31.4	25.4	0.0
6	72.6	11.0	84.3	88.9	66.6	177.6	0.0	0.0
7	52.9	10.0	87.6	73.8	0.0	86.0	4.3	181.4
8	174.4	27.1	47.5	62.2	16.8	28.3	0.0	154.2
9	26.2	0.0	27.2	48.5	40.1	33.4	0.0	222.2
10	68.9	63.0	48.0	46.3	98.8	86.9	52.6	66.8
11	80.6	88.0	170.3	16.1	108.4	67.7	83.4	58.8
12	49.6	50.5	97.8	40.5	69.8	19.8	34.7	71.7
13	299.6	0.0	131.9	39.8	91.0	61.0	80.8	34.2
14	34.2	47.8	62.5	65.2	46.3	47.3	20.9	82.9
15	256.1	0.0	108.3	73.9	24.5	230.2	0.0	144.8
16	57.1	67.9	159.8	42.2	46.0	74.6	0.0	23.0
17	121.6	34.5	84.6	59.9	0.0	0.0	38.5	83.7
18	127.7	33.0	67.3	85.1	59.9	70.9	82.0	17.8
19	64.0	26.9	98.1	62.7	16.4	89.5	35.2	164.8
20	82.5	30.4	104.8	69.0	114.3	136.5	69.8	76.5
Mean	95.7	35.6	80.9	68.7	64.0	73.0	28.3	85.6
Standard deviation	72.4	25.6	40.5	33.3	47.8	56.6	30.8	70.5
Cohen's dz	0.94512		0.32600		0.17061		0.93602	
Statistical power	**0.97398**		0.27128		0.10888		**0.97163**	

Supplementary Table S7. Statistical analysis of experiments 1-6 by a generalised linear model (GLM) assuming pseudo-binominal error structure and logit link function. The proportion of time spent by differently treated females in the pheromone treated field of the olfactometer was used as response variable. treatment as fixed factor and the total time spent in both fields (pheromone plus control) as weights. If a significant effect was found in an experiment, selected key treatments were pairwise compared. The exact description of the different treatments is given in Fig. 1 of the main paper. The original data for each experiment are given in Supplementary Tables S1-6.

Figure	Exp. no.	Compared Treatments	*F*	*p*
2A-B	1	1A-B	8.4572	**0.006115**
2C-F	2	2A-D	6.2913	**0.0007414**
		2A vs. 2B	14.049	**0.001285**[#]
		2A vs. 2C	10.209	**0.00281**[#]
3A-E	3	3A-E	6.8472	**6.828E-05**
		3A vs. 3B	7.3193	**0.01006**[#]
		3A vs. 3C	19.086	**0.0002122**[#]
3F-H	4	4A-C	11.721	**5.752E-05**
		4A vs. 4B	24.993	**0.00002828**[#]
		4A vs. 4C	13.506	**0.0007493**[#]
4A-C	5	5A-C	4.3277	**0.01779**
		5A vs. 5C	4.4844	**0.04118**[#]
		5B vs. 5C	8.7031	**0.010698**[#]
4D-F	6	6A-D	15.226	**5.703E-06**
		6A vs. 6C	6.0333	**0.01914**[#]
		6A vs. 6D	28.045	**0.000018**[#]
		6C vs. 6D	12.114	**0.00195**[#]

[#]Benjamini-Hochberg corrected for multiple comparisons
